# Utilizing deliberative engagement for identifying implementation strategy priorities: lessons learned from an online deliberative forum with dental professionals

**DOI:** 10.1186/s43058-023-00496-2

**Published:** 2023-09-21

**Authors:** Inga Gruß, Tim Dawson, Charles D. Kaplan, Daniel J. Pihlstrom, Jeffrey L. Fellows, Deborah E. Polk

**Affiliations:** 1 Center for Health Research, Kaiser Permanente Northwest, 3800 N. Interstate, Portland, OR 97227 USA; 2 The Art of Democracy, LLC. 51 Roycroft Avenue, Pittsburgh, PA 15228 USA; 3 Sunrise Community Counseling Center, 537 S. Alvarado St, Los Angeles, CA 90057 USA; 4 Permanente Dental Associates, 500 NE Multnomah St #100, Portland, OR 97232 USA; 5 School of Dental Medicine, University of Pittsburgh, 3501 Terrace Street, Pittsburgh, PA 15260 USA

**Keywords:** Deliberative engagement, Guideline implementation, Dentistry, Lessons learned

## Abstract

**Background:**

Selecting effective implementation strategies to support guideline-concordant dental care is a complex process. We are drawing on data collected during the DISGO study to reflect on barriers we encountered in implementing a deliberative engagement process for discussing implementation strategies relevant to the evidence-based guideline targeted in this intervention. The goal is to identify factors that may influence the success of deliberative engagement as a technique to involve healthcare staff in identifying priorities for implementation strategies.

**Methods:**

We drew on online chat transcripts from the deliberative engagement forums collected during the DISGO study. The chat transcripts were automatically generated for each discussion and captured the written exchanges between participants and moderators in all participating dental clinics. Chat transcripts were analyzed following a content analysis approach.

**Results:**

Our findings revealed barriers to the successful implementation of deliberative engagement in the context of the DISGO study. Participants were not familiar with the materials that had been prepared for the forum and lacked familiarity with the topic of deliberation. Participants also did not share divergent viewpoints and reinforced existing ideas rather than introducing new ideas.

**Conclusions:**

In order to ensure that obstacles that were encountered in this study are not repeated, it is important to carefully consider how staff can effectively be prepared for the deliberations. Participants must be familiar with the content of the guideline, and most questions about the content and evidence should be answered before the deliberative engagement sessions. If perspectives among staff on a guideline are homogenous, briefing materials should introduce perspectives that complement existing views among staff. It is also necessary to create an environment in which staff are comfortable introducing opinions that may not be held by the majority of colleagues.

**Trial registration:**

This project is registered at ClinicalTrials.gov with ID NCT04682730. The trial was first registered on 12/18/2020. https://clinicaltrials.gov/ct2/show/NCT04682730.

Contributions to the literature

- This article reflects on the use of deliberative engagement for selecting clinical practice guideline implementation strategies
- The findings suggest that there may be unique challenges for leveraging deliberative engagement in this context
- Recommendations to preventing and addressing possible challenges are provided

## Background

Engaging healthcare professionals in determining priorities and pathways for implementation of quality improvement initiatives and evidence-based practice guidelines is considered important for closing the research to practice gap [1–4]. Participatory approaches that have been utilized include discrete choice experiments [5], community-based approaches [6], Delphi panels [7] and concept mapping [8]. Using such approaches to involve healthcare professionals in the implementation of novel practices can, however, be challenging [9]. To address challenges and have methods for a variety of organizational settings and implementation scenarios, expanding the methodological tool kit for engaging healthcare professionals is important.

For this analysis, we drew on data collected as a part of the DISGO study^1^ to reflect on barriers encountered implementing a deliberative engagement process among dental professionals for discussing implementation strategies. Deliberative engagement processes, also referred to as deliberative democracy, are processes in which citizens become involved in public decision-making by deliberating on a given issue and informing decision-making of governing bodies [10]. In the context of health care, priorities of several interest groups representing different hierarchical may collide when determining priorities for service improvement initiative [11]. Establishing connections between these interest groups to reflect different priorities and gather support for different perspectives may establish support for improved implementation of initiatives [11]. In deliberative engagement, recommendations from participants are shared with decision-makers to inform their decision-making process and establish greater degrees of legitimacy for policy decisions [10]. Deliberative engagement has been used to engage the public in determining priorities for health goals [12], policy discussions [13, 14], and cancer screening decisions [15] in the context of healthcare. What made implementation of deliberative engagement in this context novel, was the involvement of healthcare staff – rather than the public – in deliberative engagement and the online mode of delivery. An important reasons for selecting this participatory approach was the goal of testing a large-scale small-group engagement with limited demands on participants' time.

The goal of the present analysis is to identify barriers to the successful implementation of deliberative engagement in the DISGO study. This deliberative forum was held online. We are identifying barriers that may apply to other studies attempting to engage healthcare professionals in online deliberations. This analysis can support the decision making process of others who may be considering the use of deliberative engagement as a participatory approach with healthcare professionals in the context of implementation. Barriers and reflections are presented side-by-side to present and discuss the data in an engaging way.

## Methods

### Setting

The DISGO study—a stepped-wedge, cluster randomized trial—took place in the Kaiser Permanente Dental Program (KP Dental) that is part of the Kaiser Permanente Northwest (KPNW) integrated health care system. The goal of the DISGO study was to improve adherence to the pit-and-fissure dental sealant guideline by increasing placement rates of therapeutic sealants [16]. The pit-and-fissure guideline had been introduced at KP Dental for the first time in 2008. At the time, no implementation activities accompanied the roll-out of the guideline. The guideline provides recommendations about the placement of preventive and therapeutic sealants: Preventive sealants are foremost placed on intact occlusal (biting) surfaces of molars of children and adolescents, while therapeutic sealants are placed on occlusal surfaces to arrest incipient caries [17]. Preventive sealants had been the focus of previous implementation initiatives at KP Dental, and the DISGO research study focused on therapeutic sealants; adherence to the recommendations of the guideline regarding the placement of therapeutic sealants was low at KP Dental [18].

All staff members including all clinical roles (general dentists, pediatric dentists, orthodontists, dental hygienists, expanded function dental assistants (EFDA), and nurses) as well as administrative roles such as front office staff and office managers from 16 dental clinics identified for the intervention were invited to participate in deliberative engagement sessions as reflecting principles of inclusion foundational to deliberative theory and practice [19]. During a 15-min prerecorded presentation prepared by the research team, all staff members were provided with an introduction to deliberative engagement, a summary of the content of the pit-and-fissure guideline, data summarizing KP Dental’s and each respective clinic’s placement rate of therapeutic sealants, a summary of barriers to adherence, and implementation strategies meant to address these barriers. Participants were provided the opportunity to ask questions at the time of the presentation as well as to share questions afterwards with the research team. The information regarding the content of the guideline, adherence rates and implementation strategies was summarized in a workbook that participants received right after the 15-min presentation (see Polk 2021 for an excerpt of the workbook). Workbooks serve to provide background information to ensure that all participants in a deliberative forum can draw on a comparable body of knowledge about a given topic and introduce new perspectives on a given topic [20].

Four weeks later, participants were invited to a 90-min, moderated online forum discussion on the Common Ground for Action platform.^2^ Moderators were professional facilitators trained to ensure that all participants—regardless of role or position in the organizational hierarchy—had the opportunity to contribute to the deliberations. During this discussion, they were able to exchange views about barriers and implementation strategies in a written chat. Finally, they completed a survey immediately upon completion of the discussion to share their views and opinions about relevant implementation strategies. The survey results were summarized and reported to dental leadership.

All participants received an information sheet that provided elements of consent. A waiver of written consent had been obtained. Participants did not receive an incentive for participation in the study, and all activities took place during work hours. The study was approved by the Kaiser Permanente Northwest Institutional Review Board. The DISGO study had null findings; these results are explored elsewhere [18].

### Analytical approach

For this article, we drew on chat transcripts of the deliberative forums to arrive at a comprehensive understanding of barriers to introducing an online deliberative engagement process into the context of selecting implementation strategies in dental offices. The chat transcripts were automatically generated for each discussion and captured the written exchanges between participants and moderators.

The chat transcripts were analyzed by an experienced qualitative researcher using a directed content analysis approach [21]. A directed content analysis approach is guided by combining predefined codes created based on theoretical assumption with codes that emerge during the coding process as relevant for capturing underlying patterns in the data. The final coding dictionary included eight codes (see Appendix 1 for all codes and definitions). The coding dictionary was used to code all 31 chat transcripts that had been selected randomly. From the coded text segment, themes were derived that related to the research question.

## Results and discussion

During the deliberations, we encountered several challenges to the deliberation of implementation strategies. We discuss each of the barriers and challenges, possible explanations, and reflect on how these may be overcome in future deliberations that focus on the discussion of guidelines and implementation strategies.

### Staff members were not familiar with the workbook and often were uncertain about the focus of the deliberations

The deliberative forums had been organized around the content of the workbook, which was referenced throughout the discussion by the facilitators. Staff had had the opportunity to learn about the topic of the deliberative forum on several occasions (see methods section above). The assumption that participants would be familiar with the workbook was the starting point for the deliberations. This, however, proved inaccurate.

Many participants voiced confusion about the workbook and its content. “This is the first time I’m hearing about a workbook,” or “I did not get a workbook, so I’m already off to a great start” were phrases shared by participants. As a result, many staff members were uncertain about the focus of the deliberations: “What is the topic about? I know it’s sealants but not exactly sure what.” [EFDA] The lack of knowledge of the focus of the deliberations left many participants ill-prepared to productively participate in the deliberations. There are several possible explanations for this finding as well as suggestions for preventing a similar challenge in the future.

### Participants did not make/have time to read the workbook

Several participants mentioned during the discussions that they did not have time to read the workbook and felt ill-prepared: “We don’t get time to read workbooks,” a hygienist said and a dentist said: “I feel like more preparation would have benefitted this group prior to this exercise.” Effective management and translation of technical knowledge such as provided in the workbook has been documented as a key challenge to successful deliberation [22]. Possible strategies to overcome this challenge could include engaging participants in longer discussions about the technical information prior to the deliberations rather than relying on them reading the workbook individually [22]. If carving out additional time to bring researchers and participants together beforehand could be challenging, the research team could try to provide staff members with protected time before the forums to read the workbooks. This time was not provided in the context of the DISGO study. Negotiating protected time may be challenging in an organizational setting where healthcare staff may follow tight schedules or guidelines by labor unions establish allocation of work time.

### Participants lacked motivation to prepare for the engagement forum

Another possible explanation for why participants had not read the workbook could be a lack of motivation to participate in the forum. All staff members—including clinical as well as administrative staff—at each of the selected dental clinics had been invited to participate in the activity. Participation was scheduled during work hours when all staff members would otherwise have convened for an all-staff meeting. Attendance rates were high across all forums (363 staff members attended, 377–379 had been expected), but high attendance did not translate into active participation in all forums.

When deliberations take place in civic spaces, many invitees decline invitations [23]. Those who participate may be motivated by the possibility of influencing political decision-making, learning about perspectives of others, and the opportunity to immerse themselves in a new environment [24]. In the context of the DISGO study, participants were determined by the research team; all staff members affiliated with the clinics selected for the intervention had been strongly encouraged to participate. Therefore, providing incentives for participants to be engaged before and during the forum in this context may be necessary.

Highlighting the opportunity for staff to influence organizational decision-making about implementation strategies and possible workflow changes may be one way to incentivize active participation if participation is required. Participation could also be voluntary. Motivating staff to participate may also be accomplished by clearly articulating the relevance of a guideline to a given context before the deliberations. This could be done by highlighting immediate and distal outcomes such as fewer patients requiring restorations, or, public health implications such as contributing to caries prevention.

### Participants were still learning about the content of the guideline

Comments by staff showed that they were uncertain about the evidence behind the guideline and its recommendations. During a phase of formative research, participants had questioned placing sealants on incipient lesions [25]. These doubts also re-emerged during the deliberative forums: “The ADA guideline recommends sealants on sound and incipient occlusal caries with a sound occlusal surface. But we were all trained to remove decay… I think the paradigm has shifted and a lot of us aren't comfortable with it.” [Dentist].

Yankelovich has proposed seven stages of public judgement—going from dawn of awareness (stage 1) to making a responsible moral and emotional decision (stage 7) [26]. Deliberation is mostly appropriate in later stages of this process. Quotes that show that participants were still in the process of engaging with the evidence of the guideline suggest that participants may have benefited from additional information and training about the guideline before entering deliberations. Learning opportunities that had been created in the context of this study were not sufficient to clearly explain the guideline and its evidence to all staff members.

### Participants shared narrow perspectives on the guideline and readily supported each other’s claims and opinions

The transcripts also revealed that the forums served to share misinformation about the guideline and its recommendations [27]. Staff members expressed doubts about the veracity of the evidence and the appropriateness of placing therapeutic sealants. One dentist said that “any caries present is a deal breaker for some providers” and an EFDA said that “I can't count how many times on an xray the doctor said oh this is real shallow and started to open the tooth and it was much bigger…into the DEJ and putting a sealant over caries just goes against everything I've ever been taught/told.” When staff members expressed doubts about therapeutic sealants, other staff members did not question their statements or introduced alternative perspectives. In contrast, staff members generally supported and praised each other’s opinions. This was observed across all forum discussions. Rather than engaging participants in an exchange of a wide variety of perspectives on the guideline, the deliberations served to provide a platform for proliferation of perspectives that were not rooted in evidence.

### Lack of diverse perspectives on the guideline

Deliberative engagement rests on the assumption that participants exchange perspectives on a given topic, sharing their diverse points of view and reasoning for holding their perspectives to learn from each other [28]. Group deliberations where participants with like-minded perspectives exchange opinions are referred to as enclave deliberations [29]. Previous research on enclave deliberations has shown that even in like-minded groups, participants’ opinion can shift towards the center, although less so than in groups where more diverse perspectives are present [30]. Introducing information that is not already held by group members—for example through workbooks—is important for this to happen [30].

Based on Role Theory [31], the research team had assumed that different professional roles would hold different perspectives on the guideline. Thus, diversity of perspectives was defined in relationship to professional roles. Chat transcripts suggested that this assumption was not justified. There were few differences in opinion across roles. Since participants in the DISGO study were largely unfamiliar with the workbook, during the deliberations, groups could only could draw on knowledge already held by group members. As these perspectives were limited, overall the deliberations did not succeed in diversifying participants’ perspective on the guideline.

To prevent enclave deliberations, it is important to ensure that new perspectives – either through briefing materials or by including participants who bring different perspectives to a deliberation – are introduced that can be taken into considerations by participants. We previously discussed strategies to increase the likelihood that participants will engage in self-study of the workbook.

### No mechanisms were built into the forums to prevent the spread of misinformation

The deliberations at times inadvertently supported the spread of misleading information about the guideline and its evidence. There was no mechanism built into the forum to prevent colleagues from propagating and spreading misinformation. By design, forum facilitators were not content experts, but experts in facilitating deliberative engagements. This was in line with standard protocols for deliberative forums [32]. In the context of civic forums, experts may be present to be able to provide answers to content questions participants may have [32].

In the context of the DISGO study, where 61 deliberative forum discussions were held online over 8 months, it would not have been feasible to have content experts available consistently. Content experts also are not meant to be actively involved in the deliberations to correct participants’ perspectives but rather are meant to be available as a resource to draw on [32]. This again highlights the importance of engaging staff members in deliberations related to guidelines they are familiar with to ensure that deliberations do not support the spread of misinformation or contradict evidence provided in a given guideline.

### Deliberations among staff members may not facilitate drawing out divergent perspectives

The willingness to readily support each other’s points of view—rather than contribute additional perspectives—could be related to the fact that deliberative forums in organizational contexts bring together colleagues rather than strangers (as is the case in forum discussions in civic spaces). Teams made up of healthcare professionals working in the same clinical specialty typically share similar educational backgrounds and professional values. It has been argued that such teams may exhibit convergence in thinking that promotes group cohesiveness akin to groupthink that can result in poor decision making [33]. Beyond the relative homogeneity of the groups brought together, all participants also were in ongoing professional relationships with each other. Sharing alternative perspectives during a one-time deliberative session may cause friction for their ongoing work relationship. Ensuring that staff members are comfortable voicing opposing viewpoints is central for successfully holding deliberative engagement forums in an organizational context.

## Conclusion

Our findings describe barriers to the successful implementation of deliberative engagement with healthcare professionals during the DISGO study and provide suggestions how these barriers may be addressed in future studies. It is important to keep in mind that our analysis was based on one study only. The goal of this analysis is to inform decision-making and planning processes of other researchers who are considering utilizing deliberative engagement.

In conclusion, deliberative engagement forums may be an appropriate tool in implementation if researchers are able to ensure that the following conditions are met. Future organizers need to carefully consider how they can effectively engage staff in preparations for the deliberative forums and which mechanisms are available to ensure that staff have time to learn about an upcoming forum, its focus, and the evidence behind a guideline. Participants must be familiar with the content of the guideline, and most questions about the content and evidence have been answered before the deliberative engagement sessions. The presence of an expert panel could also be considered to ensure that questions about evidence can be answered during the forum discussions. If perspectives among staff on a guideline may be homogenous, workbooks need to clearly introduce perspectives that complement existing views. It is also crucial to create an environment in which staff are comfortable introducing opinions that may not be held by the majority of colleagues.

## Data Availability

The qualitative data analyzed for the current study are not publicly available due to them containing information that could compromise research participant privacy; the codebook is available upon request.

## Appendix

### Appendix 1. Coding dictionary

Forum confusion: Any expressions that capture that participants do not know what is going on in the forum, including:

- statements that they are confused
- asking if everyone should participate
- requests for clarification
- questions about the workbook
- questions about goal of the forum
- questions about terminology related to the platform

Promotive voice: Any expression of ways to improve existing work practices and procedures, including.

- benefits of new/suggested strategies that may lead to improvement
- new behaviors which may be beneficial to the clinic
- improvements to existing procedures (in response to materials received)

Prohibitive voice: Any expression of participants’ concern about existing practices, behaviors, barriers, suggestions and opinions, including.

- reasons why a strategy may not be appropriate or feasible
- problems with workflows or suggestions
- expressing dissenting views on opinion of others
- critical questions about workflows, issues, suggestions

Agreement: Any statements supporting positions/statement made by others or the status quo, including.

- acceptance of existing workflows
- acknowledgement that current practices work
- supporting opinions of others
- willingness to try out some of the suggested strategies

(Critical) reflections: Any statements that capture thoughts about existing procedures and the deliberative democracy process, including.

- reasons why barriers from workbook may not be correct
- suggestions how something could be interpreted differently
- pointing out low agreements with strategies
- providing nuance on when or how a strategy may work
- reasons for existing practices
- pros and cons of deliberative democracy forum

Deferral: Deferral to others, to powers beyond themselves in determining workflows, including.

- statements that participant is not making any decisions
- statements that others are responsible for guideline implementation
- comments that say this is not relevant to professional practice of participant

Other barriers: Any statement that describe alternative barriers, including.

- debates of what constitutes a barrier
- listing of additional barriers

Sealant confusion: Any statements that indicate that interviewee/participant continues to confuse preventative and treatment sealants.

## Abbreviations

DISGO: Dissemination and Implementation of Sealant Guidelines in Organizations
EFDA: Expanded function dental assistant
KP: Kaiser Permanente
KP Dental: Kaiser Permanente Dental
KPNW: Kaiser Permanente Northwest

## Footnotes

1 DISGO stands for **D**issemination and **I**mplementation of **S**ealant **G**uidelines in **O**rganizations.

2
https://www.nifi.org/en/cga-online-forums
